# Metabolic performance and feed efficiency of black soldier fly larvae

**DOI:** 10.3389/fbioe.2024.1397108

**Published:** 2024-04-30

**Authors:** Niels Thomas Eriksen

**Affiliations:** Department of Chemistry and Bioscience, Aalborg University, Aalborg, Denmark

**Keywords:** *Hermetia illucens*, growth models, metabolic models, substrate conversion efficiency, net growth efficiency

## Abstract

The black soldier fly (BSF), *Hermetia illucens*, is used in entomoremediation processes because its larvae can use a variety of organic residues with high efficiency. However, feed efficiencies are variable and characterized by uncertainties. Recently developed growth and metabolic performance models have predicted across different studies that BSF larvae have used 53%–58% of the feed components they have assimilated, in terms of carbon equivalents, for growth throughout their lifetime when reared on chicken feed. This is termed their average net growth efficiency. The remainder of the carbon has been lost as CO_2_. However, mass balances made under similar conditions show that the weight gained by BSF larvae corresponds to only 14%–48% of the feed substrates removed, indicating substrate conversion efficiency. Both performance indicators show even greater variability if more feed substrates are considered. Feed assimilation and growth rates, costs of growth, maintenance, and larval lifespan have been shown to affect how efficiently BSF larvae convert feed into growth. The differences between average net growth efficiencies and substrate conversion efficiencies further indicate that feed is often not used optimally in entomoremediation processes and that the overall yield of such processes is not determined by larval performance alone but is the result of processes and interactions between larvae, substrates, microbes, and their physical environment. The purpose of this study is to illustrate how quantification of the metabolic performance of BSF larvae can help improve our understanding of the role of the larvae in entomoremediation processes.

## 1 Introduction

The black soldier fly (BSF), *Hermetia illucens*, is an insect species used in entomoremediation processes. It can degrade waste, valorize organic residues, and be used to produce feed, food, and fertilizer (see, e.g., Wang and Shelomi, 2017; Gligorescu et al., 2020; Beesigamukama et al., 2023). During its life cycle, the BSF passes through a larval phase of five instars, a prepupal and a pupal phase, and finally the reproductive adult phase (De Smet et al., 2018). Only the larvae grow and take up feed to any greater extent. Newly hatched larvae can develop into prepupae in as little as 13 days (Chia et al., 2018) and reach live weights of up to approximately 300 mg. Often, however, they take longer to develop without reaching this weight. After they transform into prepupae, they lose weight (Georgescu et al., 2020; Eggink and Dalsgaard, 2023). BSF larvae and prepupae normally contain approximately 50% protein and 30% fat, with the fat content being most variable (Eriksen, 2022). The larvae are reared mixed with their feed substrate, as microbes commonly are, and can thus be viewed as catalysts and products. Thereby, entomoremediation processes share characteristics with animal husbandry and microbial biotechnology.

Feed efficiency, that is, the efficiency by which BSF larvae use and convert organic residues into their own biomass, is highly important and one of the main arguments for rearing BSF larvae. Different performance indicators are used to describe and compare feed efficiencies in BSF larvae and livestock in general. Common performance indicators (some studies use alternative naming) are the bioconversion rate (ratio of total increase in larval weight to weight of the feed substrate provided), substrate conversion efficiency (ratio of total increase in larval weight to weight of the substrate removed during the process), the substrate conversion ratio (inverse of the substrate conversion efficiency), and the substrate reduction rate (ratio between weights of the lost and supplied feed substrate). It is difficult to directly measure feed intake and excretion of fecal pellets in BSF larvae and, hence, assess feed efficiencies as they live mixed in their substrates and frass. Most often, the performance indicators are therefore quantified via mass balances, where the weights of larvae and residual feed substrate plus frass at the time of harvest are compared to the initial weights of the starter larvae and the supplied feed substrate. The performance indicators vary considerably when BSF larvae are reared on different feed substrates. They are affected by concurrent microbial activity in the feed substrates (Bekker et al., 2021; Zhang et al., 2023), and some are sensitive to the level of inorganic and indigestible components in the feed (Klammsteiner et al., 2021). The substrate conversion efficiency, which is the one most closely related to the performance of the larvae, ranges between 0 and 0.35 for most feed substrates (see, e.g., Bosch et al., 2019; Siddiqui et al., 2022). Chicken feed, which has been used as a base-line substrate in several studies on BSF larvae, provides a selection of comparable performance data. Despite this being an excellent feed substrate for BSF larvae, there has still been three-time differences between the highest and lowest substrate conversion efficiencies in chicken feed, with values ranging from 0.14 to 0.48, respectively (Table 1).

**TABLE 1:** Performance indicators of BSF larvae reared on chicken feed or similar feeds and typically harvested when a fraction of the larvae had reached the prepupal stage. Bioconversion rate, BR; substrate conversion efficiency, SCE; substrate conversion ratio, SCR; substrate reduction rate, SRR. Stage is the predominant developmental stage; larvae, L; or prepupae, P, at the time of harvest. *X*_max*,WW*_ and X_*max,DW*_ are the maximal wet weight or maximal dry weight of larvae or prepupae, respectively. Δ*X*_*DW*_ is the difference in larval dry weight from start until harvest. Δ*W*_*DW*_ is the difference between the dry weights of the supplied feed substrate and residues at harvest. W_DW,0_ is the dry weight of the supplied feed substrate.

Feed	*X*_max*,WW*_	*X*_max*,DW*_	BR ∆XDWWDW,0	SCE ∆XDW−∆WDW	SCR −∆WDW∆XDW	SRR −∆WDWWDW,0	Source
	mg	mg					
Chicken feed	-	33.9	-	0.23	-	-	Oonincx et al. (2015)
Chicken feed	251	-	0.13	*0.15*	-	0.85	Lalander et al. (2019)
Hen feed	229	-	-	0.27	-	-	Bava et al. (2019)
Chicken feed	-	55.6	0.21	0.32	-	0.68	Gold et al. (2020)
Chicken starter mash	148.4	-	0.18	*0.35*	-	0.50	Broeckx et al. (2021)
Chicken feed	-	69	-	0.34	-	-	Veldkamp et al. (2021)
Broiler feed	216.2	-	-	0.48	-	0.44	Addeo et al. (2021)
Chicken feed	-	66	-	0.38	-	0.66	Klammsteiner et al. (2021)
Chicken feed	-	-	*0.08*	*0.16*	-	-	Naser El Deen et al. (2023)
Chicken feed	*191*	-	-	*0.27*	*3.75*	*0.64*	Eggink et al. (2023)
Chicken feed	*80*	-	-	0.14	-	0.43	Rossi et al. (2023)
Chicken feed	-	-	0.18	*0.41*	*2.4*	*0.44*	Damanik et al. (2024)

Numbers in italics are either read from graphs using the software available at https://www.graphreader.com/ or SCE calculated based on the BR, SCR, and SRR.

The actual performance of the BSF larvae can be hard to decipher from the performance indicators in Table 1. They pay no attention to the growth phase of the larvae, despite the fact that this phase determines the overall process outcome. Some studies have examined the overall metabolic flows and processes, feed assimilation, growth, and respiration across most of the lifespan of BSF larvae (Bekker et al., 2021; Laganaro et al., 2021; Hansen et al., 2023). The combined outcome of these flows and processes has been referred to as their metabolic performance (Laganaro et al., 2021). Larval feed efficiency can then be evaluated by an alternative performance indicator, the net growth efficiency (NGE), which depends solely on the metabolic performance of the larvae. Often, NGE is not only used to describe the ratio between energy stored in animals and energy assimilated via their feed but also to describe the rate of carbon equivalents built into BSF larvae, *r*_*X*_, compared to the rate of carbon equivalents assimilated by the larvae, *r*_*A*_, as follows:

$$\text{NGE}=\frac{r_{X}}{r_{A}}.$$ (1)

Eq. (1) describes the instantaneous performance of the larvae. The growth and feed assimilation rates can be integrated across the larval lifespan to provide information on their overall performance, from when they are introduced into their feed substrate and until they are harvested.

$${\text{NGE}}_{\text{avg}}=\frac{\int r_{X}}{\int r_{A}}=\frac{X-X_{0}}{A}.$$ (2)

*X*_*0*_ and *X* represent larval weights at start and at harvest, respectively, and *A* is the total amount of assimilated feed components. To express the variables in Eqs 1 and 2 in terms of carbon equivalents, it has been assumed that the elementary composition of the larvae as well as their feed corresponds to the average composition of living organisms (Laganaro et al., 2021). NGE_avg_ differs from the feed conversion efficiency since the denominator in Eq. (2) considers only the feed that has been assimilated and entered the metabolism of the larvae to be used either for growth (the numerator in Eq. (2)) or catabolized to provide energy for biosynthesis and other life-sustaining functions in the larvae. *A* cannot be determined from mass balances since some of the assimilated carbon is lost as CO_2_.

The purpose of this study is to illustrate how determining the metabolic performance of BSF larvae can help improve our understanding of their role in entomoremediation processes, discuss how the metabolic performance of BSF larvae has been addressed, compare substrate conversion efficiencies to net growth efficiencies, evaluate how efficient BSF larvae really are, and advocate for increased attention to the processes taking place while the BSF larvae grow and do their job in entomoremediation processes.

## 2 Kinetic growth and metabolic performance models

**FIGURE 1:**
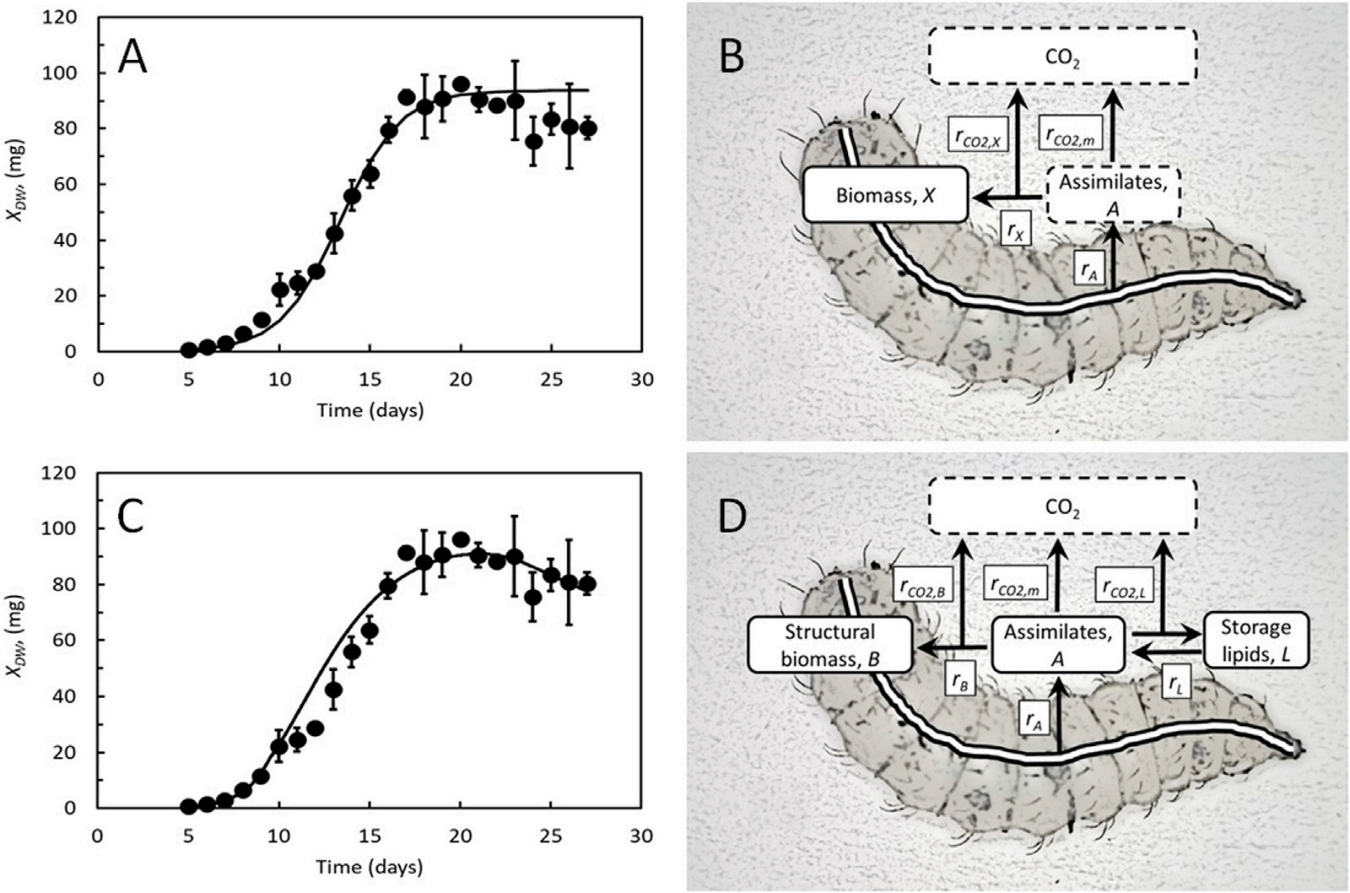
**(A)** Best fit of the Verhulst logistic equation to the measured DW of BSF larvae reared on chicken feed. Data from Laganaro et al. (2021). **(B)** Key metabolic flows and processes considered in the kinetic growth model by Laganaro et al. (2021). Feed components are assimilated at rate, *r*_*A*_, or used for growth at rate, *r*_*X*_, and metabolized to generate energy for growth or maintenance at rates *r*_*CO2,X*_ and *r*_*CO2,m*_, respectively. **(C)**: Best fit of the dynamic growth model by Eriksen (2022) to the same data as in Panel A, using the following parameters: maximal specific feed assimilation rate, *a*_max_ = 1.2 day^−1^, cost of growth, *Y* = 0.44, and maintenance, *m* = 0.08 day^−1^. **(D)**: Key metabolic flows and processes considered in the dynamic growth model by Eriksen (2022). Feed components are assimilated at rate, *r*_*A*_, and used for growth of structural biomass or storage lipids at rates *r*_*B*_ and *r*_*L*_, respectively, or metabolized to generate energy for growth, synthesis of storage lipids, or maintenance at rates *r*_*CO2,B*_, *r*_*CO2,L*_, and *r*_*CO2,m*_, respectively. Data are partly adopted from Eriksen (2022).

The amount of BSF larvae produced from a given amount of assimilated feed will depend on the metabolic flows and processes in the larvae, from when they are introduced into their feed substrate and until they are harvested. The metabolic flows and processes depend on the size and age of the larvae, and metabolic models combining kinetics and stoichiometry can provide theoretical frameworks for measurements of the larvae. Verhulst’s logistic equation and other sigmoidal functions closely resemble measured growth curves of BSF larvae when reared on different feeds and under different conditions (Pamintuan et al., 2019; Sripontan et al., 2020; Bekker et al., 2021; Laganaro et al., 2021; Matheka et al., 2021; Knudsen et al., 2022; Hansen et al., 2023). The increase in larval weight is close to exponential during the first half of their lifespan, meaning that their weight specific growth rate is almost constant and close to maximal. Thereafter, growth slows and finally stops when the larvae reach their maximal weight and enter the prepupal stage. To evaluate the metabolic performance of BSF larvae, Laganaro et al. (2021) combined the kinetics of Verhulst’s logistic model with expressions for feed assimilation and CO_2_ production, as shown in Figures 1A,B. Growth is considered the rate-limiting process, and the larvae assimilate feed to grow and produce energy. The energy is used to convert feed components into the molecules that are incorporated into a new tissue (cost of growth) and to sustain the basic functions of life not associated with growth (maintenance). CO_2_ is generated from the metabolism associated with the cost of growth and maintenance. The metabolic part of the model is like the general model for growth and maintenance of microorganisms (Pirt, 1982), and the release of CO_2_ is like the classical model for microbial lactic acid production (Luedeking and Piret, 1959). Growth and CO_2_ production rates together represent the feed assimilation rate, which is difficult to determine experimentally in complex feed substrates. More details can be found in Laganaro et al. (2021).

Three studies have evaluated the metabolic performance of BSF larvae reared at different conditions, each revealing insights that could not have been obtained solely from mass balance studies. In the first study, Laganaro et al. (2021) found, in agreement with former studies (Lalander et al., 2019), that degassed sludge is a poor substrate for BSF larva growth. Slower growth, smaller prepupae, and prolonged developmental times were observed when the degassed sludge fraction in the feed substrate was increased from 0% to 75%. Larval CO_2_ production also increased in a dose-dependent manner, showing that they assimilated feed at the same or even at higher rates as they did with chicken feed despite their slower growth. Maintenance and cost of growth were, however, increased by the degassed sludge, forcing the larvae to use more of their feed for other purposes, leading to slow growth.

In the second study, Bekker et al. (2021) found, as observed before (Cheng et al., 2017; Palma et al., 2018; Chen et al., 2019), that substrate moisture content affects BSF larva growth. Small larvae grew fastest in the driest substrates, but they also grew for the shortest period, and it was the larvae in the most wet substrates that achieved the highest weight. However, no differences were found in the cost of growth or maintenance in substrates with moisture contents between 45% and 75%. Conversely, the microbial CO_2_ production in the substrates was highly dependent on the substrate moisture content. It exceeded that of the BSF larvae in the driest substrates to such an extent that microbial substrate utilization probably restricted the feed availability for the larvae. Thus, the moisture content of the substrate appeared to mainly affect the competition for food between BSF larvae and microbes, which subsequently affected the larvae most in the driest substrates where microbes were most active.

In the third and final metabolic performance study (Hansen et al., 2023), BSF larvae assimilated feed and grew at similar rates on starch-deprived brewery waste as on starchy chicken feed, though the larvae formed prepupae with lower weights when brewery waste was included. The cost of growth and maintenance were similar on both feed substrates, supporting that BSF larvae are indeed highly adaptable in their nutritional needs (Bonelli et al., 2020). Other studies have found quite variable growth patterns of BSF larvae reared on brewery waste (Jucker et al., 2019), which thus seems to be a resource of uneven quality for BSF larvae and, furthermore, can be affected by microbial pretreatment (Gebiola et al., 2023). Metabolic performance studies on BSF larvae are still too few in number to provide a coherent picture of how larval metabolism is affected by substrate composition or variations in physical conditions. One take-home message from the three metabolic performance studies is that the weight and growth patterns of BSF larvae are affected by a complex set of environmental conditions, and so is NGE_avg_. The latter, and thus also the feed efficiency in entomoremediation processes, is affected at least by feed assimilation and growth rates, costs of growth and maintenance, and lifespan of the larvae.

## 3 Dynamic growth and metabolic performance models

The model in Figures 1A,B describes growth as the rate-limiting metabolic process, but at least in some insect larvae, feed assimilation is the rate-limiting metabolic process (Woods and Kingsolver, 1999). Padmanabha et al. (2020) presented a dynamic model that linked growth to feed uptake in BSF larvae. The specific feed assimilation rate (feed assimilation rate relative to the weight of the larvae) is highest in newly hatched larvae and decreases to 0 in the prepupa, following a logistic (sigmoidal) function as the larvae approach their maximal weight. Thereby, this model ends up describing sigmoidal growth curves, closely resembling those of BSF larvae. These principles have been included in a second metabolic performance model for BSF larvae (Figures 1C,D), where lipid content was also included as a variable (Eriksen, 2022). BSF larvae increase their fat content until they become prepupae and then lose fat and weight (Liu et al., 2017; Beniers and Graham, 2019; Eggink and Dalsgaard, 2023). The model operates with two rate-limiting metabolic flows or processes: the feed assimilation rate and the rate of production of structural biomass (larval tissues except storage lipids). Both rates decrease logistically. However, the specific growth rate only decreases after the larvae reach instar 5. This is consistent with observations in BSF larvae (Eriksen, 2022) and other insects (Shingleton et al., 2008), causes an imbalance between feed assimilation and growth, and leads to an excess of feed assimilates that are converted into storage lipids. Since lipids are normally more reduced than feed, their synthesis also results in CO_2_ production which maintains redox neutrality. When the larvae reach the prepupal stage, feeding stops, the storage lipids are remobilized to cover the energy needs for maintenance, and the weights of the larvae decrease. This model is a simple differential energy budget model (Kearney, 2021) that excludes maturation and reproduction, which are primarily relevant to later life stages (Maino and Kearney, 2015). More details can be found in Eriksen (2022).

**FIGURE 2:**
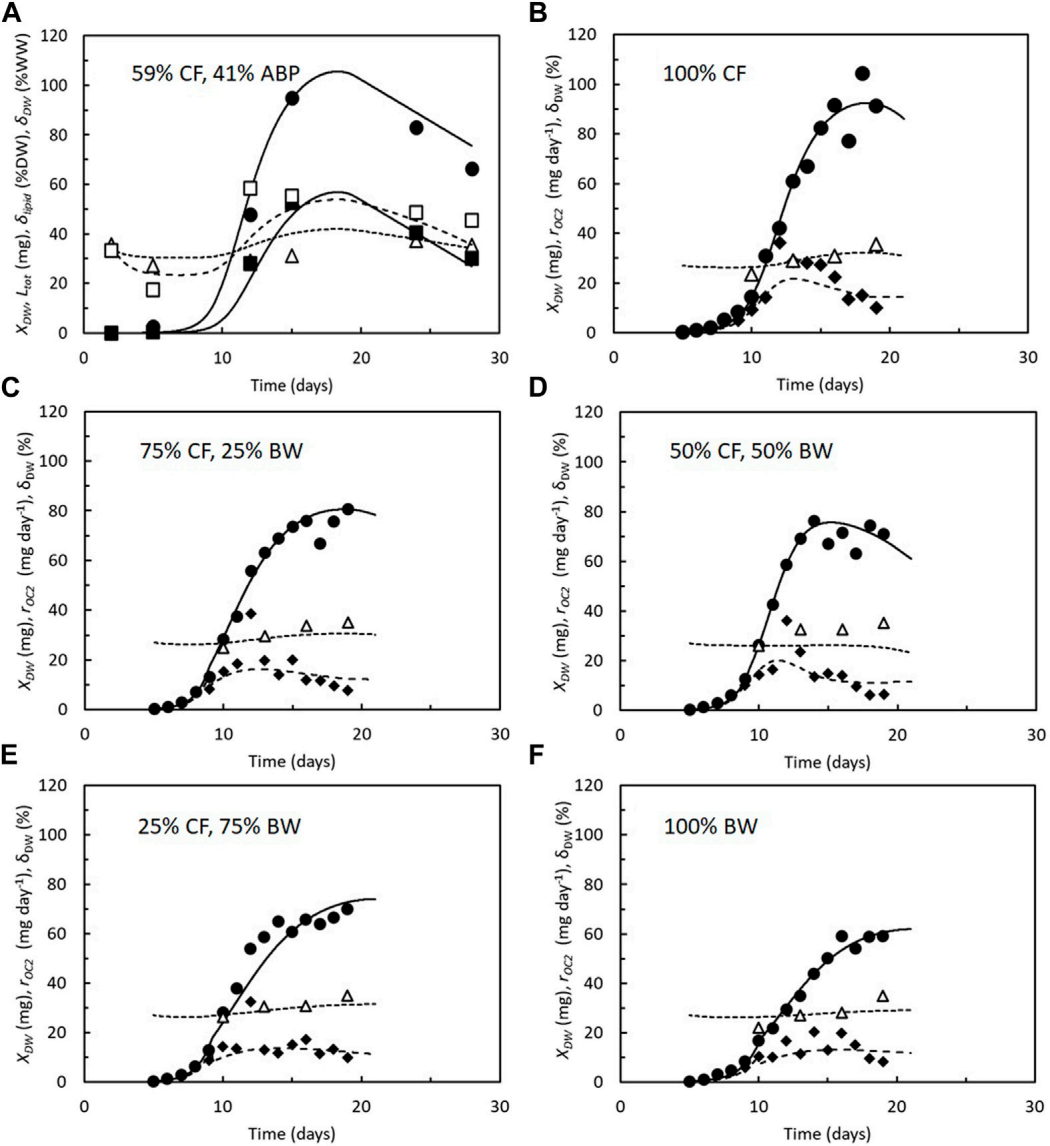
Dry weight, *X*_*DW*_ (●, solid line); CO_2_ production rate, *r*_*CO2*_ (♦, dashed line); total lipid content, *L*_*tot*_ (_▀_, solid line); relative lipid content, *δ*_*lipid*_ (Δ, dashed line); and dry weight content, *δ*_*DW*_ (Δ, dotted line), of BSF larvae reared on **(A)** chicken feed (CF) mixed with 41% agricultural by-products (ABP). Lines represent the model (Figure 1B) fitted to *X*_*DW*_ and *L*_*tot*_ with the following parameters: *a*_max_ = 1.5 day^−1^ and *Y* = 0.44, m = 0.08 day^−1^. A model of predictions of *δ*_*DW*_ used to verify the coherence of the model. Data from Eggink and Dalsgaard (2023) and model parameters from Laganaro et al. (2021). **(B–F)** Mixtures of chicken feed (CF) and brewery waste (BW). Data points represent an average of three replicate larval cultures. Lines represent the model (Figure 1B) fitted to *X*_*DW*_ and *r*_*CO2*_ with the following parameters: maximal specific feed assimilation rate, *a*_max_ = 1.2–1.5 day^−1^; cost of growth, *Y* = 0.35–0.44; and maintenance, *m* = 0.10–0.13 day^−1^. Model predictions of *δ*_*DW*_ are used to verify the coherence of the model. Data and model parameters are adopted from Hansen et al. (2023).

The model (Figures 1C,D) has provided coherent descriptions of larval dry weight, CO_2_ production rate, lipid content, and dry weight content (indirect measures of larval lipid content), matching the four experimental datasets that were available in 2022 (Liu et al., 2017; Beniers and Graham, 2019; Bekker et al., 2021; Laganaro et al., 2021). Two newer studies from 2023 also provide coherent measurements of larval dry weights with either lipid content (Eggink and Dalsgaard, 2023) or CO_2_ production rate (Hansen et al., 2023) of BSF larvae reared on different mixtures of chicken feed and organic residues. The results of these studies are also well-reproduced (Figure 2). Thus, the model seems to reflect the overall metabolic flows and processes of BSF larvae, despite the metabolism being segregated into just four metabolic flows and processes and the tissue composition structured into just two compartments. Some discrepancy between both models (Figures 1B,D) and experimental data, particularly at the time the larvae reached approximately 20% of their maximal weight (Figures 1A,C), is because neither model considers that feed intake and growth are likely paused during molting (Kivelä et al., 2020).

## 4 Feed efficiency of BSF larvae

The dynamic growth model in Figures 1C,D was published, which included a spreadsheet version for simulation of growth and key metabolic processes of BSF larvae (Eriksen, 2022), where rates of growth, *r*_*X*_, and feed assimilation, *r*_*A*_, are calculated throughout the larvae’s growth phase. An extended version of this model is included as Supplementary Material, which estimates the overall net growth efficiencies from Equation 2 and allows model outputs to be visually compared to experimental data. It should be noticed that *r*_*X*_ and *r*_*A*_ are calculated from a model that considers the metabolic flows and processes to be unbalanced and the biochemical composition of the larvae to be variable. Therefore, carbon and energy will not necessarily be conserved with the same efficiency in the larvae. In this study, these overall carbon net growth efficiencies are therefore marked by an asterisk, NGE*_avg_.

Table 2 shows NGE*_avg_ calculated for the BSF larvae represented by the six datasets mentioned above, which (presumably) include all the coherent measurements of larval dry weight in combination with either the CO_2_ production rate, lipid content, or dry weight content available today. All studies included chicken feed as a feed substrate, either by itself or in combination with other organic residues. In BSF larvae reared on a diet consisting solely of chicken feed, NGE*_avg_ was between 0.53 and 0.58, according to simulations. This means that 53%–58% of the carbon taken up and used by the larvae remained in their bodies when they were harvested. Substrate moisture content seems not to affect NGE*_avg_ to any greater degree. When different organic residues were mixed into chicken feed, NGE*_avg_ becomes more variable, with values varying from 0.26 to 0.58. Brewery waste alone resulted in an estimated NGE*_avg_ of 0.44. The main reason for NGE*_avg_ being low in brewery waste is that it took the larvae longer to reach the prepupal phase on this feed substrate than on chicken feed, and they gained less weight (Hansen et al., 2023). Their longer lifespan thus made them invest a larger proportion of their resources in maintenance. The lowest NGE*_avg_ has been seen in feed substrates rich in degassed sludge due to high maintenance rates and cost of growth (Laganaro et al., 2021).

**TABLE 2:** Overall carbon net growth efficiencies, NGE*_avg_ in BSF larvae, aged 1–5 days and until the estimated time of prepupae formation, calculated using Eq. 2, with rates of growth and feed assimilation calculated using the differential energy budget model in Figures 1C,D. Number of different feed substrates, *n*.

Feed	*n*	NGE*_avg_	Data source
Chicken feed	1	0.56^a^	Liu et al. (2017)
Chicken feed	1	0.57^a^	Beniers and Graham (2019)
Chicken feed	1	0.53^a^	Laganaro et al. (2021)
25%–75% chicken feed and 25%–75% degassed sludge	3	0.26–0.46^a^	Laganaro et al. (2021)
Chicken feed, 45%–75% substrate moisture content	4	0.53–0.58^a^	Bekker et al. (2021)
Chicken feed	1	0.53^b^	Hansen et al. (2023)
25%–75% chicken feed and 25%–75% brewery waste	3	0.48–0.53^b^	Hansen et al. (2023)
Brewery waste	1	0.44^b^	Hansen et al. (2023)
59% chicken feed, 19% rye, 12% rapeseed cake, and 10% sugar beet pellets	1	0.58^b^	Eggink and Dalsgaard (2023)

^a^ Model (Figures 1C,D) fitted to the data using the parameters given in Eriksen (2022).

^b^ Fit between model and data shown in Figure 2.

All NGE*_avg_ values measured on larvae reared on chicken feed (Table 2) are higher than the substrate conversion efficiencies measured on similar substrates (Table 1). Although these performance indicators are based a bit differently on ratios of either carbon equivalents or dry weights, NGE*_avg_ marks the upper limit of the substrate conversion efficiency. The differences between these two performance indicators indicate that feed utilization is not always optimized in cultures of BSF larvae and that it represents a potential scope for optimizing substrate conversion efficiencies, thus supporting that co-occurring microbes can also remove considerable amounts of substrates in entomoremediation processes (Bekker et al., 2021; Zhang et al., 2023). BSF larvae thus perform more efficiently, having higher feed efficiencies than indicated by the performance indicators calculated from mass balances.

## 5 Concluding remarks

A favorable feed efficiency on a variety of feedstocks is one of the main arguments for employing BSF larvae in entomoremediation processes, but even on an excellent substrate such as chicken feed, measured substrate conversion efficiencies are highly variable (Table 1) and considerably lower than estimated net growth efficiencies (Table 2). Thus, the overall outcome of entomoremediation processes is lower than optimal and seems to be determined not solely by the match between substrate composition, the nutritional needs of the larvae, and their digestive capacity but also is the result of processes and interactions among larvae, substrates, microbes, and their physical and chemical environment, from the larvae being introduced into the feed substrate and until they are harvested. More attention to the metabolic performance of the BSF larvae during growth can help elucidate their roles in complex environments and thereby provide an insight that can hardly be obtained from mass balances alone. Hopefully, this article has illustrated and inspired what such studies can be used for.
